# Development of the TP53 mutation associated hypopharyngeal squamous cell carcinoma prognostic model through bulk multi-omics sequencing and single-cell sequencing

**DOI:** 10.1016/j.bjorl.2024.101499

**Published:** 2024-09-02

**Authors:** Ying Zhang, Yue Cui, Congfan Hao, Yingjie Li, Xinyang He, Wenhui Li, Hongyang Yu

**Affiliations:** The Second Affiliated Hospital of Harbin Medical University, Department of Radiation Oncology, Harbin, China

**Keywords:** Hypopharyngeal squamous cell carcinoma, Prognosis, TP53 mutation, Single-cell sequencing

## Abstract

**Objective:**

The aim of this study was to construct a prognostic model based on the TP53 mutation to calculate prognostic risk scores of patients with HPSCC.

**Methods:**

TP53 mutation and transcriptome data were downloaded from the TCGA databases. Gene expression data from GSE65858, GSE41613, GSE3292, GSE31056, GSE39366, and GSE227156 datasets were downloaded from the GEO database. GSEA, univariate, multivariate Cox analyses, and LASSO analysis were employed to identify key genes and construct the prognostic model. ROC curves were utilized to validate the OS and RFS results obtained from the model. The associations between risk scores with various clinicopathological characteristics and immune scores were analyzed via ggplot2, corrplot package, and GSVA, respectively. Single-cell sequencing data was analyzed via unbiased clustering and SingleR cell annotations.

**Results:**

Initially, two key genes, POLD2 and POLR2G, were identified and utilized to construct the prognostic model. Samples were divided into different risk groups via the risk scores obtained from the model, with high-risk group samples exhibiting poorer prognosis. Furthermore, the risk score exhibited a positive correlation with lymphatic metastasis in patients and the immune scores of CD4^+^ T, CD8^+^ T, dendritic cell, macrophage, and neutrophil. The immune responses also exhibited notable disparities between the high- and low-risk groups. The results of single-cell sequencing analysis demonstrated that epithelial cells and macrophages were relatively abundant in HPSCC samples. POLD2 and POLR2G exhibited higher expressions in epithelial cells, with most of the identified pathways also enriched in epithelial cells.

**Conclusion:**

The prognostic model exhibited a significant capacity for predicting the prognosis of HSPCC samples based on the TP53 mutation conditions and may also predict the cancer characteristics and immune infiltration scores of samples via different risk scores obtained from the model.

**Level of evidence:**

Level 5.

## Highlights

• The HPSCC prognostic model of TP53 mutation was constructed via POLD2 and POLR2G.
• Risk scores from the model were associated with cancer conditions and immune infiltration.
• Higher expression cells of POLD2, POLR2G, and metabolism pathways were analyzed.

## Introduction

Hypopharyngeal carcinoma is a head and neck malignancy with a relatively low incidence. However, its prognosis is poor,^1^, ^2^, ^3^ with the 5-year overall survival rate is only 30%–35%.^4^, ^5^ Hypopharyngeal carcinoma poses additional challenges to treatment strategies due to its tendency to metastasis.^6^

Approximately 95% of hypopharyngeal carcinomas are Hypopharyngeal Squamous Cell Carcinomas (HPSCC) which are classified as Head and Neck Squamous Cell Carcinomas (HNSCC) and exhibit the distinctive characteristics of squamous epithelial carcinoma.^3^ A large proportion of HNSCCs are tightly associated with mutations in the tumor suppressor gene TP53. The reported TP53 gene mutation rate in HNSCC is 70.4%, with the highest mutation rate of 83.5% in laryngeal and hypopharyngeal cancers.^7^ It has been shown that mutations in the TP53 gene can significantly affect the prognosis of HNSCC.^8^ Furthermore, the TP53 mutation was likewise confirmed to be associated with the immune response.^9^ Despite the advancements in comprehending the mutations within the TP53 gene were acquired,^10^ the majority of research has focused only on HNSCC rather than malignant alterations in any specific region of the head and neck, for instance, HPSCC. Thus, there is a significant necessity to conduct a comprehensive investigation of the TP53 gene mutation in HPSCC with the aim of accurately predicting the prognosis influence brought by TP53 mutations and identifying the potential key therapeutic site for achieving precise treatment of HPSCC in the future.

The rapid development of high-throughput omics sequencing techniques and the extensive application of bioinformatics technology extremely provide convenience in key gene identification and predictive model establishment of diseases.^11^, ^12^, ^13^ Single-cell sequencing analysis has distinctive advantages in the comprehensive investigation of molecular expression patterns associated with a disease.^14^, ^15^ In this work, we constructed the prognostic model of TP53 mutation in HPSCC and validated its predictive stability. Moreover, the differently expressed patterns of key genes in various cells were also investigated via the single-cell sequencing analysis. Eventually, the associations between risk scores and immune infiltration were investigated and indicated the significance of TP53 mutation in tumor immuno-microenvironment. The findings in our work may provide an effective prognostic model in the prognosis prediction of HPSCC and assist in deeply comprehending the TP53 mutation mechanism in HPSCC.

## Methods

### Data source

The mutation and transcriptome data of HNSCC were obtained from The Cancer Genome Atlas (TCGA) database, as well as the transcriptome data of HNSCC in GSE65858, GSE41613, GSE3292, GSE31056, and GSE39366 dataset and the single-cell transcriptome sequencing data of hypopharyngeal cancer in GSE227156 dataset from Gene Expression Omnibus (GEO) database. Additionally, we retrieved 41 metabolic pathways from MSigDB.

### Prognostic model construction using TCGA-HNSCC samples

The mutant gene was initially extracted from the HNSCC samples obtained from TCGA, followed by preprocessing of the transcriptome data and clinical information data, resulting in a total of 528 samples being obtained. Subsequently, a total of 471 samples with both genomic and transcriptome sequencing data were obtained after eliminating missing values, which comprised 316 samples with TP53 mutation and 155 samples lacking TP53 mutation. Then, the Gene Set Enrichment Analysis (GSEA) software (V4.2.2) was then employed for pathway enrichment analysis to identify the significant pathways (*p*-values < 0.05) and associated genes. These genes were immediately analyzed via univariate Cox analysis (survival package V3.5-7), multivariate Cox analysis, and least absolute shrinkage and selection operator (LASSO) analysis (glmnet package V4.1-8). Ultimately, a prognostic model was constructed utilizing key genes identified via the aforementioned analyses and the forest plot analysis was conducted using the forestplot package (V3.1.1).

### Validation in independent datasets

Based on the above downloaded datasets of HNSCC, clinical information data and expression data matrix were generated. The pROCpackage (V1.18.4) was employed for generating Receiver Operating Characteristic (ROC) curves. The survivminerpackage (V0.4.9) was utilized for plotting survival curves to validate the model. GSE65858, GSE41613, GSE3292, and GSE31056 datasets were employed for Overall Survival (OS) analysis, while the GSE39366 dataset was used to conduct Recurrence Free Survival (RFS) analysis.

### Exploration of the association between risk scores and clinicopathological characteristics

The ggplot2 package (V3.4.3) was employed to generate violin plots illustrating the associations between risk score and various factors including gender, Tumor Node Metastasis classification (TNM) stage, clinical stage, age, tumor status, and TP53 mutation status. The Differentially Expressed Genes (DEGs) analysis between high-risk and low-risk groups was conducted utilizing limma package (V3.57.7). Furthermore, enrichment analysis of DEGs was conducted using the GOplot package (V1.0.2).

### Immune scores analysis

The immune cell scores of HNSCC samples were calculated using TIMER 2.0 (timer.cistrome.org), and the correlations between immune scores and risk scores were determined using the corrplot package (V0.92). Subsequently, a total of 29 immune-related gene sets, including the immune cells, functions, and pathways were constructed. Subsequently, the gene sets scores in high-risk and low-risk groups were quantified using the GSVA package (V1.49.4).

### Single-cell sequencing data analysis

The data analysis of single-cell sequencing was conducted utilizing the R package (V4.3.1). Specifically, we initially selected the tumor data in the GSE227156 dataset, and the expression matrix was processed using Seurat V5 (https://satijalab.org/seurat; https://www.biorxiv.org/content/10.1101/2022.02.24.481684v1.full). Cells with UMI < 1000 or UMI > 20000, number of measured genes <300 or >5000, and mitochondrial percentage exceeding 50% were strictly excluded in the procedure of quality control. Subsequently, the standard Seurat procedure was performed for standardization and normalization, followed by batch effect removal using the Harmony integration method. Unbiased K-NearestNeighbor (KNN) clustering was performed, with the cluster with 0.3 resolution selected for subsequent analysis. The cell annotation was performed via the R package SingleR (V2.3.5). The scoring of the key metabolic gene sets was conducted utilizing the AddModuleScore function from the Seurat package. The VlnPlot and DimPlot functions from the same package were employed for data visualization.

### Statistical analysis

All analyses were performed using R language (version 4.3.0), with *p-*values less than 0.05 considered statistically significant.

## Results

### Development of the prognostic model for TCGA-HNSCC samples

**Fig. 1:**
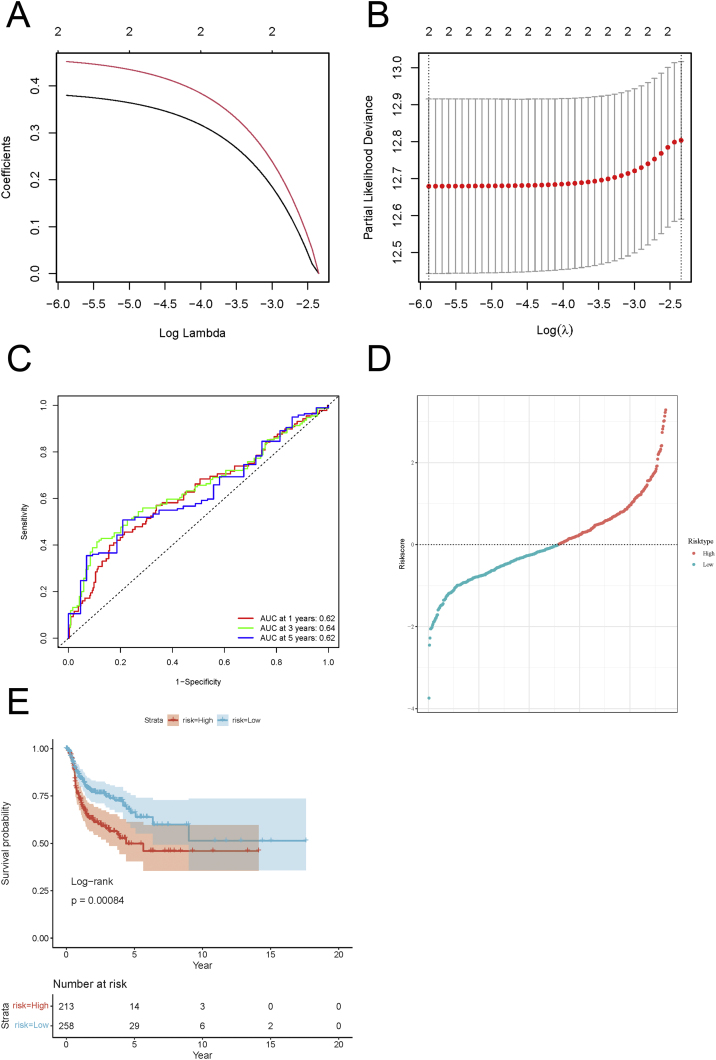
Construction and assessment of the prognostic model. (A) Path maps of regression coefficients of LASSO; (B) Cross-validation curves of LASSO; (C) ROC curves of the prognostic model in 1-year, 3-year, and 5-year survival durations. (D) Risk type group distribution based on risk scores. (E) Survival curves of the high-risk and low-risk groups and comparative analysis of survival durations between the high-risk and low-risk groups. LASSO, Least Absolute Shrinkage and Selection Operator.

Initially, the GSEA was conducted for a total of 41 pathways related to metabolism obtained from the transcriptomic data of HNSCC samples from TCGA (Supplemental Fig. 1). Following the filter for key pathways via *p*-values < 0.05, a total of 7 pathways were selected, containing a total of 307 associated genes. Subsequently, univariate and multivariate Cox analysis, as well as LASSO analysis, were performed for the aforementioned genes. As exhibited in Fig. 1A and B, we ultimately identified two key genes, POLD2 and POLR2G, upon which we constructed a prognostic model by multiplying the regression coefficient obtained from multivariate Cox regression analysis with the normalized expression level of each metabolic gene as follows: Risk score = 0.391 * POLD2 + 0.464 * POLR2G. After model construction, the ROC curves of 1 year, 3 years, and 5 years (Fig. 1C) were generated to observe its predictive capacity. Furthermore, the samples were divided into low-risk and high-risk groups based on the median risk score derived from the TCGA cohort (Fig. 1D), samples in the high-risk group presented significantly worse prognostic conditions than those in the low-risk group. Then the survival curves of these two groups were plotted and the comparative analysis between them was performed. As depicted in Fig. 1E, the patients in the low-risk group may have longer survival durations and higher survival probability.

### Validation of the model using independent datasets

**Fig. 2:**
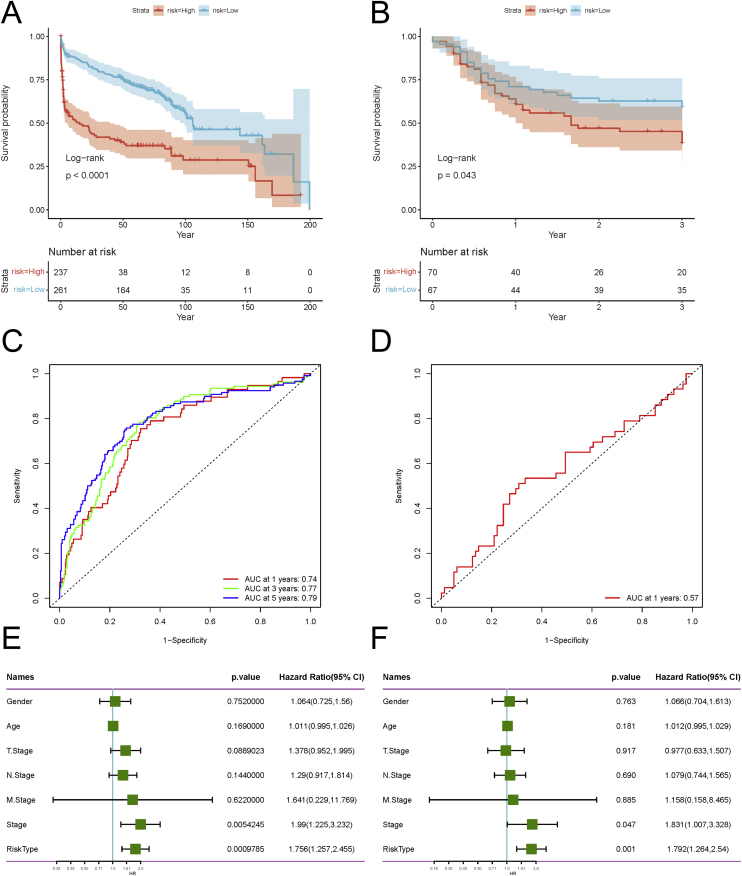
Validation of the prognostic model. (A) OS curves of the prognostic model validation. (B) RFS curves of the prognostic model validation. (C) ROC Curves for validation of the prognostic model in 1-year, 3-year, and 5-year OS prediction. (D) ROC Curves for validation of the prognostic model in 1-year RFS prediction. (E) Results of univariate Cox regression analysis in patient characteristics. (F) Results of multivariate Cox regression analysis in patient characteristics. OS, Overall Survival; RFS, Relapse-Free Survival; ROC curve, Receiver Operating Characteristic curve.

With the aim of validating the robustness of the prediction model we developed, we employed four independent GEO datasets, encompassing GSE65858, GSE41613, GSE3292, and GSE31056, to merge into a combined validation set to perform the OS analysis and utilized the GSE39366 dataset for the RFS analysis. All the analyses were proceeded between the low-risk and high-risk groups, which obtained similar results (Fig. 2A and B). The ROC curves for OS at 1 year, 3 years, and 5 years were simultaneously plotted and the AUCs of them were also found to be high, with values of 0.74, 0.77, and 0.79 (Fig. 2C). Likewise, the AUC for RFS at 1 year was as high as 0.57 (Fig. 2D). Subsequently, univariate and multivariate Cox regression analyses were performed again to determine whether the model could independently predict outcomes, with *p-*values less than 0.05 considered significant. As illustrated in Fig. 2E and F, the results of the above analyses demonstrated the outstanding predictive capability of the prognostic model in predicting external data. The predictive prognostic model we developed can possess universal applicability.

### Association between risk scores and clinicopathological characteristics

**Fig. 3:**
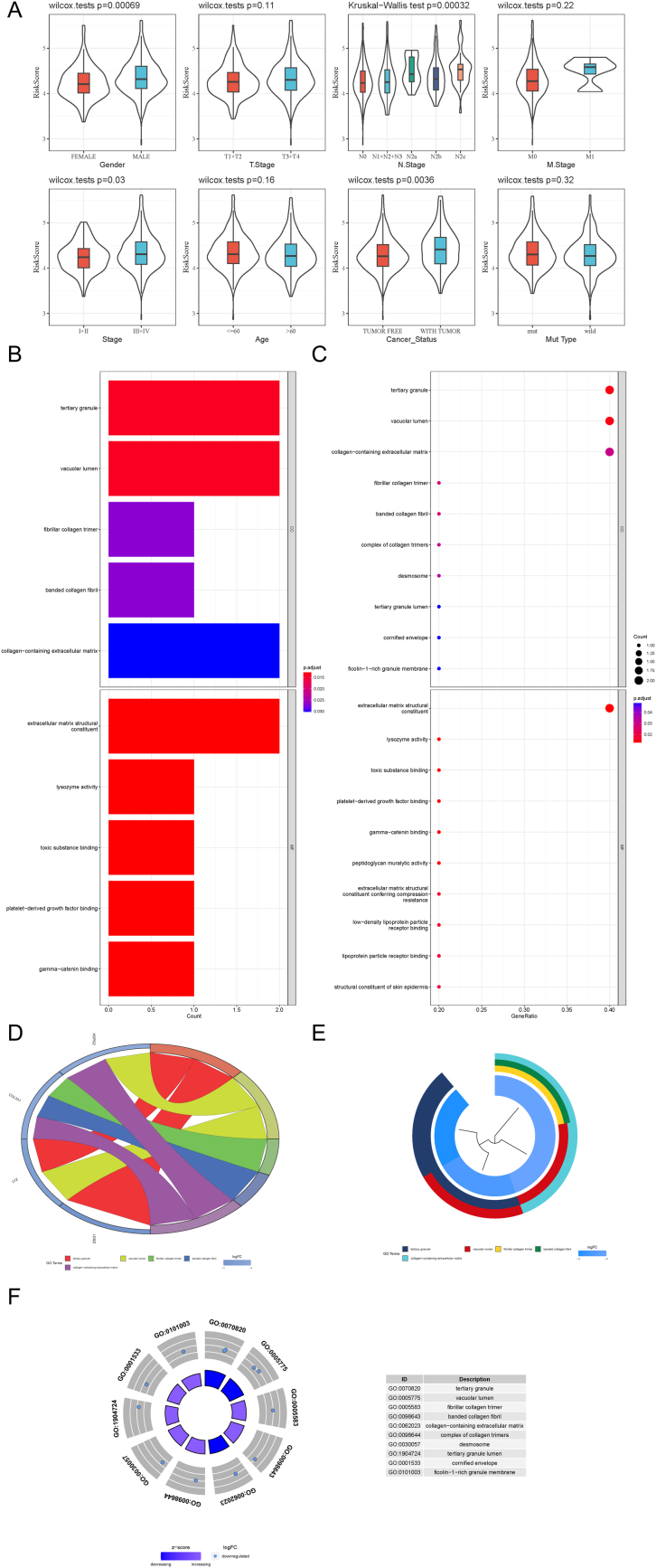
The association between risk scores and clinicopathological characteristics and GO analysis of DEGs. (A) The associations between risk score and gender, TNM stage, clinical stage, age, cancer status, and TP53 mutation status. (B) Histogram of GO enrichment in DEGs between the high-risk and low-risk groups. (C) Dot plot of GO enrichment. (D) Chord plot of GO enrichment. (E) Clustering diagram of GO enrichment. (F) Circle plot of GO enrichment. GO, Gene Ontology; TNM stage, Tumor Node Metastasis stage; DEGs, Differentially Expressed Genes.

As depicted in Fig. 3A, the associations between risk score and various characteristics including gender, TNM stage, clinical stage, age, cancer status, and TP53 mutation status in the TCGA-HNSCC samples were illustrated via violin plots and the results indicated that the risk score exhibited a positive correlation with the presence of lymphatic metastasis in patients.

### Enrichment analysis of DEGs between different risk score groups

Subsequently, we identified the DEGs between the low-risk and high-risk groups and performed the Gene Ontology (GO) analysis. The results exhibited in Fig. 3B‒F manifested that the DEGs were mainly enriched at the tertiary granule and vacuolar lumen and the molecular function was primarily involved in extracellular matrix structural constituent.

### Association between risk scores and immune system

**Fig. 4:**
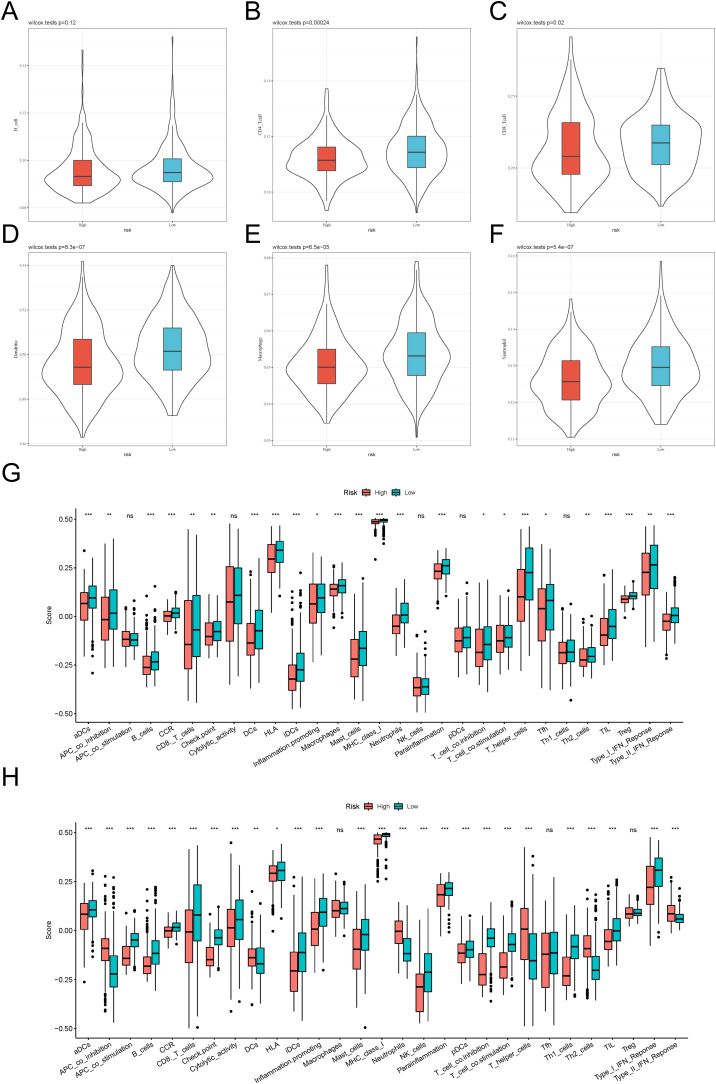
The association between risk scores and the immune system. (A–F) Violin plots of the associations between risk score groups and B-cell, CD4^+^ T-cell, CD8^+^ T-cell, dendritic, macrophage, and neutrophil. (G) Box plot of the associations between risk score groups and 29 immune-related gene sets in TCGA-HNSCC data. (H) Box plot of the associations between risk score groups and 29 immune-related gene sets in the combined validation set. TCGA-HNSCC data, Data of Head and Neck Squamous Cell Carcinoma obtained from The Cancer Genome Atlas.

In order to investigate the immune microenvironment of tumor tissue, we performed score analysis for six types of immune cells on TCGA data in combination with TIMER2. As depicted in Fig. 4A‒F, five out of six types of immune cells, including CD4^+^ T-cell, CD8^+^ T-cell, dendritic cell, macrophage, and neutrophil, exhibited significant differences between the low-risk group and the high-risk group, with the neutrophil exhibited the most significant disparity. Broadly speaking, samples in the low-risk group may own more powerful immune responses than those in the high-risk group. We then constructed a total of 29 immune-related gene sets mainly involved in the immune cells, functions, and pathways. The scores of high-risk and low-risk groups were quantified using GSVA in the TCGA-HNSCC data and merged GEO datasets (GSE65858, GSE41613, GSE3292, GSE31056). As depicted in Fig. 4G and H, there were a total of 24 and 26 gene sets exhibiting statistically significant differences between the low-risk group and the high-risk group in the TCGA-HNSCC data and merged GEO datasets, respectively. Among all the different gene sets, Major Histocompatibility Complex (MHC) Class 1 exhibited the highest scores in both TCGA and GEO datasets, which may indicate a closer association between MHC1 and HNSCC.

### Single-cell sequencing data analysis

**Fig. 5:**
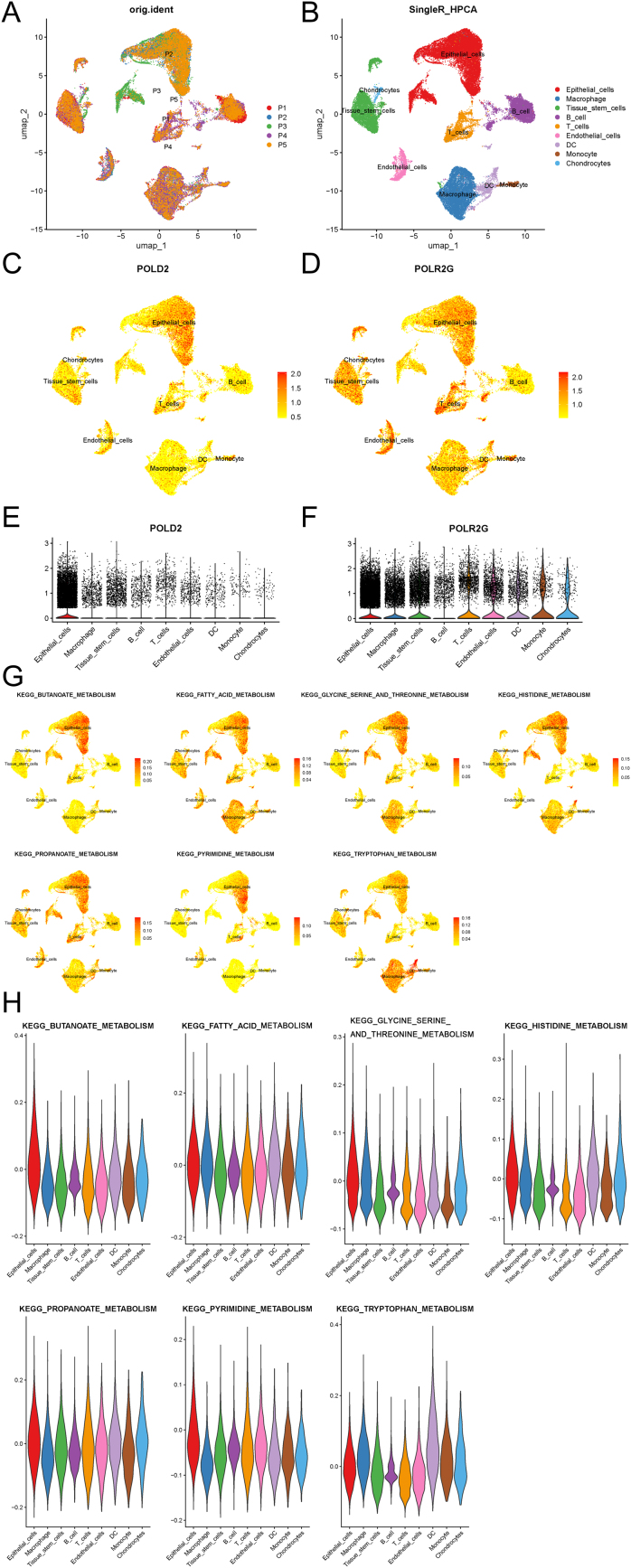
Single-cell sequencing data analysis. (A) Samples distribution after batch effect removal using Harmony. (B) Cell types distribution obtained using unbiased clustering and SingleR cell annotations. (C and D) Dot plots of the expression levels of key genes POLD2 and POLR2G in different cells. (E and F) Violin plots of the expression levels of key genes POLD2 and POLR2G in different cells. (G) Dot plots of the expression levels of key pathways in different cells. (H) Violin plots of the expression levels of key pathways in different cells.

Firstly, we performed batch effect removal on single-cell sequencing data obtained from diverse patient samples with hypopharyngeal carcinoma (Fig. 5A). Subsequently, unbiased clustering and SingleR cell annotations were performed, whose results indicated that the higher proportion of cells in samples were epithelial cells and macrophages (Fig. 5B). The expression levels of key genes in different cells were then explored, which demonstrated that POLD2 and POLR2G exhibited the highest expressions in epithelial cells, while POLR2G was highly expressed in other types of cells, encompassing macrophages, tissue stem cells, T-cells, endothelial cells, Dendritic Cells (DC), and B cells (Fig. 5C‒F). We further investigated the expression location of pivotal metabolism pathways. The results exhibited that various metabolism pathways were highly expressed in different cells, yet most of the pathways we explored showed higher expressions in epithelial cells, which may be robustly associated with aberrant proliferation of epithelial cells in squamous cell carcinoma (Fig. 5G and H).

## Discussion

As one kind of malignancy occurring in the head and neck region, HPSCC is known for its exceedingly poor prognosis. The reasons why it exhibits the most unfavorable prognosis can be attributed to the high probability of TP53 mutation and proclivity for metastasis. A recent research investigating the TP53 mutations in a total of 57 HPSCC indicated that the mutations were identified in 68% of patients. The 3-year disease-specific survival rate of patients with TP53 mutations displayed significantly worse compared with patients without mutations, TP53 mutation can be acknowledged as an influence factor in HPSCC prognosis.^16^ Several reports also demonstrated the impact of TP53 mutation in other kinds of HNSCC.^10^, ^17^, ^18^ Therefore, we constructed a prognostic model to endow distinct risk scores to patients with different TP53 mutation conditions and predict their prognosis. In the first place, a total of 471 samples were classified based on the occurrence of TP53 mutation, resulting in 316 samples with mutations and 155 samples without mutations. Intriguingly, 307 genes in 7 key pathways associated with TP53 mutation were identified. Subsequently, we utilized univariate, multivariate Cox analysis, and LASSO analysis, resulting in two key genes, POLR2G and POLD2, were obtained and employed to construct the prognostic model for HPSCC. After model construction, all samples from the TCGA database were incorporated into the model for acquiring the risk scores and divided into low- and high-risk groups. Samples in the high-risk group demonstrated evidently poorer OS than those in the low-risk group. These results were similarly validated using a combined validation set. Subsequently, we analyzed the relationships between risk scores with clinicopathologic features and immune cell scores, which further demonstrated the significant application value of our predictive system. Eventually, the single-cell sequencing data of hypopharyngeal cancer were analyzed with the aim of affirming the cell types and the highly expressed cells of key genes.

POLD2 and POLR2G, the genes we identified for prognostic model construction, have been confirmed to encode the catalytic subunit of DNA polymerase delta and the G subunit of RNA polymerase II, whose encoding productions were involved in the processes of DNA replication, damage repair, and DNA transcription, respectively. It was already confirmed that the occurrence of various cancers is closely linked to DNA mutations and damage repair mechanisms in which the encoding product of POLD2 also participates. Thus, the significance of the association between the occurrence and progression of cancer and POLD2 is indisputable. Several recent studies have also confirmed the high expression levels of POLD2 in the pathogenesis of various malignancies, including breast cancer,^19^ glioblastoma,^20^ multiple myeloma,^21^ and bladder urothelial carcinoma.^22^ Via bioinformatics technology, POLD2 had been demonstrated to be significantly overexpressed in most tumors and may be a molecular biomarker for pan-cancer prognosis likewise.^23^ Meanwhile, this study also confirmed that overexpression of POLD2 may be associated with a poorer prognosis of cancers, which exhibit a high degree of resemblance to the findings obtained in our study. In light of the aforementioned findings, we could infer that the expression level of POLD2 holds significant potential in the application of cancer prognosis prediction.

In comparison to the comprehensive investigation of POLD2, POLR2G received comparatively less attention in cancer occurrence and progression. There only exist a few studies that illustrated POLR2G exhibited overexpression trends in hepatocellular carcinoma.^24^, ^25^ Moreover, the expression of POLR2G had been revealed to be associated with gastric carcinoma.^26^ According to the renowned genetic central dogma, DNA transcription exhibits a pivotal process in the expression of cancer-associated genes and the promotion of cancer development, hence, we can infer that POLR2G and its encoded products play a crucial role in the pathogenesis and progression of various malignancies. Noteworthily, the finding of our current work substantiated this deduction.

The immune microenvironment exhibits a crucial role in the whole procedure of cancer occurrence and development, HNSCCs are no exception. According to the report of Chen,^27^ there were diverse kinds of immune cells involved in HNSCCs, including innate and adaptive immune cells. Furthermore, it also was illustrated that the high rate of metastases and recurrences of HNSCCs were also likely attributed to the interaction between the surrounding tissue and various immune cells in the immune microenvironment. An earlier study indicated that in the patients with Oral Squamous Cell Carcinoma (OSCC), there were abundant CD8^+^ T-cells around the tumor, and CD4^+^ T-cells were found to be abundant in most patients, either.^28^ Another study illustrated that higher CD4^+^ T and CD8^+^ T-cell levels highly contributed to the improvement of OS and RFS in HNSCCs,^28^ which was significantly consistent with our research findings. In our work, MHC was inferred to be closely associated with HNSCC. As far as we know, there were several reports that demonstrated that decreased expression of MHC assisted the tumor cells to evade adaptive immunity. The study of Theodoraki^29^ demonstrated approximately half of the HNSCCs exhibited lower expression of MHC class I. In addition to T-cells, macrophages were reported to be common in the HNSCCs.^30^ Recently, high levels of macrophage have been proven to be associated with lymph node metastasis and advanced stage of HNSCCs.^31^ However, the correlations between gene expressions and immune cell infiltration in multiple HNSCCs did not receive adequate attention. To the best of our knowledge, our work first illustrated the positive association between key genes (POLD2 and POLR2G) expression levels and immune infiltration in HPSCC, which may establish the preliminary foundation for further investigation into the immune infiltration and immune microenvironment of HPSCC.

The current work is the first to identify the influence of POLD2 and POLR2G in the occurrence of TP53 mutation in HPSCC. However, the scope of our research has been limited to preliminary exploration, in future investigations, it is imperative to perform experimental validation on the expression patterns of these two genes using a substantial number of clinical samples with or without TP53 mutation.

To sum up, we constructed an HPSCC prognostic prediction model with significant predictive capacity and obtained two key genes identified via several bioinformatics analyses. Furthermore, we also analyzed the highly expressed cell types of key genes and the association between the risk scores based on HPSCC TP53 mutation conditions via the model with cancer characteristics. The findings we obtained may provide new insight into the research of TP53 mutation condition in HPSCC and introduce a novel approach for prognosis prediction of HPSCC in clinical practice. Furthermore, the two key genes we identified may hold potential as therapeutic targets for the precise treatment of HPSCC in the future.

However, there are inevitably a few minor limitations in our work. Firstly, our study exclusively included publicly available data from the TCGA database and did not undergo further validation in other extensive clinical databases. Secondly, owing to the absence of immune status, nutritional status, and comprehensive clinical information in the TCGA database, we were unable to conduct a more thorough investigation into their correlation with HPSCC. Notwithstanding these limitations, our findings still carry significant implications for the clinical diagnosis and treatment of HPSCC.

## Ethics approval and consent to participate

Not applicable.

## Consent for publication

Not applicable.

## Data availability statement

The datasets used and/or analyzed during the current study are available from the corresponding author upon reasonable request.

## Authors’ contributions

Ying Zhang and Yue Cui: Conceptualization, writing-original draft. Congfan Hao and Yingjie Li: Data curation, Methodology. Xinyang He and Wenhui Li: Formal Analysis, Investigation. Hongyang Yu: Writing-review and editing.

## Funding

None.

## Conflicts of interest

The authors declare no conflicts of interest.
