# Supplementary material for: Development of the TP53 mutation associated hypopharyngeal squamous cell carcinoma prognostic model through bulk multi-omics sequencing and single-cell sequencing
Source: Braz J Otorhinolaryngol. 2024 Sep 2;91(1):101499. doi: 10.1016/j.bjorl.2024.101499 (PMC11466543; doi:10.1016/j.bjorl.2024.101499)

BJORL-D-24-00159_Supplementary Material

**Supplemental Figure 1** Gene set enrichment analysis of metabolic pathways involved in TP53 mutation of HPSCC in TCGA. TCGA, The Cancer Genome Atlas.


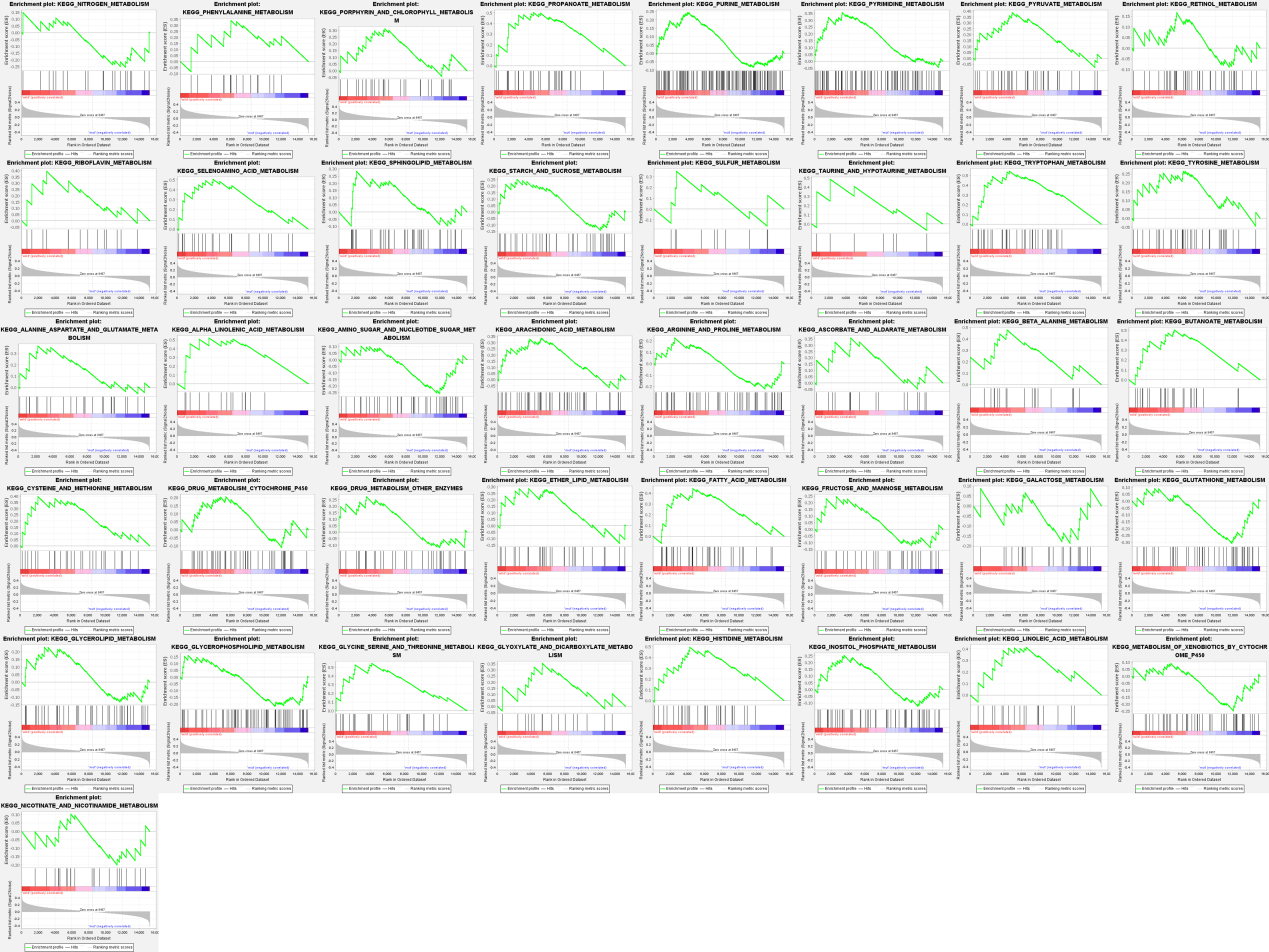

Supplement: Supplementary file 1 [file mmc1.docx]
